# SODA MAPS: A Framework for Understanding Caffeinated Sugary Drink Consumption Among Children

**DOI:** 10.3389/fnut.2021.640531

**Published:** 2021-03-10

**Authors:** Sabrina E. Halberg, Amanda J. Visek, Emily F. Blake, Kofi D. Essel, Jennifer Sacheck, Allison C. Sylvetsky

**Affiliations:** ^1^Department of Exercise and Nutrition Sciences, Milken Institute School of Public Health, The George Washington University, Washington, DC, United States; ^2^Department of Kinesiology, School of Public Health, University of Maryland, College Park, MD, United States; ^3^School of Medicine and Health Sciences, The George Washington University, Washington, DC, United States; ^4^Division of General and Community Pediatrics, Children's National Hospital, Washington, DC, United States

**Keywords:** youth, sugar-sweetened beverages, diet, caffeine, obesity, concept mapping

## Abstract

Excess sugary drink (SD) consumption is associated with childhood obesity and development of cardiometabolic disease. In addition to having high added sugar content, many SDs also contain caffeine, which may further encourage excess SD consumption among children. The objective of this study was to develop a conceptual framework of children's caffeinated SD consumption using group concept mapping, an applied social research multimethodology that collectively harnesses qualitative and quantitative data from participants to generate a visual representation of their ideas and input. Children, 8–14 years old, who reported consuming ≥12 ounces of caffeinated SDs (e.g., sodas, sweet teas) per day were recruited throughout Washington, D.C. and invited to participate. Concept mapping included three participant-driven activities: (1) brainstorming (*n* = 51), during which children reported reasons for their SD consumption, from which 58 unique reasons were identified; (2) sorting (*n* = 70), during which children sorted each of the reported reasons into categories and named each category; and (3) rating (*n* = 74), during which children rated the influence of each reason on their own caffeinated SD consumption. Similarity matrices, multidimensional scaling, and hierarchical cluster analysis were used to generate concept maps (hereafter “SODA MAPS”), which display the 58 reasons organized within eight overarching clusters. Among these eight clusters, *Taste and Feel, Something to Do*, and *Energy* were rated as particularly influential. Children's caffeinated SD consumption is encouraged not only by the palatable taste and reported preferences for these beverages (e.g., *Taste and Feel*), but also by psychological (e.g., *Mood and Focus*), biological (e.g., *Energy*), social (e.g., *Something to Do*) and environmental reasons (e.g., *Nothing Better Available*). Thus, the SODA MAPS can inform the development of tailored, multi-level SD reduction interventions that incorporate strategies to address important and currently overlooked reasons for caffeinated SD consumption among children.

## Introduction

Excess sugary drink (SD) consumption is a key contributor to excess weight gain and obesity in children (1–3). Weight gain and obesity during childhood increase the risk of multiple health issues, including type 2 diabetes (4, 5), cardiovascular diseases (6), fatty liver, and dyslipidemia (7, 8), as well as bone and joint issues (9), dental decay (10), and psychological problems (11–14). Therefore, limiting SD intake is an urgent public health priority (15).

Contrary to recommendations to limit SD intake to <8 ounces per week or to avoid SDs altogether (16), 63% of children in the U.S. drink one or more SDs per day (17). SD consumption increases with age in both girls and boys and differs by race/ethnicity and socioeconomic status (18–21), with minority and low-income populations reporting the highest SD intakes. While the palatability and accessibility of SDs are well-described reasons for SD consumption (22, 23), children's SD intake is influenced by a variety of factors, including parenting practices (22, 24, 25), nutritional knowledge (20, 26), availability of SDs at home (23), screen time (27), and fast food consumption (28).

The large quantities of added sugar in SDs are not the only cause for concern. Certain sugary drinks, such as colas and sweet teas, are also predominant contributors to caffeine intake among U.S. children (28–30). Caffeine consumption is known to elicit behavioral and psychological effects that can lead to dependence (31), and the combination of added sugar and caffeine in SDs may uniquely reinforce SD consumption behaviors among children. However, determinants specifically pertaining to caffeinated SD intake among children have not yet been studied, except with regard to energy drinks and sugary coffee beverages, which constitute only a small fraction of children's total caffeinated SD intake (32).

We recently reported physical, cognitive, emotional, and interpersonal reasons for children's caffeinated SD consumption based on qualitative data from focus group discussions with children from predominantly minority and/or low-income backgrounds (33). While these findings call attention to the complex interconnection of biological, psychological, and socio-environmental factors associated with children's SD consumption, the relative significance and interrelatedness of these reasons were not evaluated. This study, therefore, aimed to comprehensively examine multifactorial reasons for children's caffeinated SD intake using group concept mapping, an applied social research mixed methodology, which resulted in a novel, participant-driven conceptual framework, hereafter referred to as SODA MAPS. We specifically focused on children from minority and/or low-income backgrounds, who report the highest intakes of SDs and are disproportionately burdened by obesity and cardiometabolic disease (19, 34).

## Materials and Methods

### Study Design

Children, 8–14 years old participated in concept mapping, a mixed-method approach, which involves a series of participant-driven activities, including brainstorming, sorting, and rating. For brainstorming, children were recruited from pediatric primary care clinics and District of Columbia public schools. For sorting and rating, children were recruited from District of Columbia public schools, as well as afterschool programs and local community events. Depending on the location (primary care clinics and community events vs. schools and after school programs), consent forms were either given directly to parents, or students were asked to bring them home to be signed by their parent or guardian (hereafter parent). Children with signed consent forms provided assent and then were assessed for study eligibility using a brief eligibility screener questionnaire. Inclusion criteria included that the child (a) was between 8 and 14 years old; (b) consumed ≥12 ounces of caffeinated, sugary, non-diet drinks (e.g., Coca-Cola™, Pepsi™, Mountain Dew™, Arizona Iced Tea™) per day; and (c) spoke English fluently. Exclusion criteria included child-reported consumption of regular, caffeine-containing coffee, hot tea, or energy drinks (e.g., Red Bull™, Monster™) ≥1 time per week. We selected the 8-to-14-year-old age range in order to focus on children in elementary and middle school, who are less likely to consume coffee and/or energy drinks, compared with older adolescents (28).

After providing assent, participants self-reported their age, sex, and race/ethnicity and then completed the concept mapping activities. All study procedures were conducted in a pre-determined designated private space (e.g., school classroom or vacant conference room). While some participants contributed to brainstorming and also to sorting and/or rating, concept mapping methodology does not require participants to take part in all three activities (35).

All study materials were approved by the Institutional Review Boards at the George Washington University [Protocol 18091] and Children's National Hospital [Protocol 00011014]. Given the minimal time commitment required of participants for brainstorming, financial compensation was not provided; however, participants who completed the sorting and/or rating activities received a $10 gift card as compensation.

### Brainstorming

For brainstorming, each child (*n* = 51) completed the focus prompt “I drink sugar-sweetened sodas and sweet teas such as Coke^TM^, Pepsi^TM^, Mountain Dew^TM^, Dr. Pepper^TM^, and Nestea^TM^ because…” and were encouraged to list all of the reasons they could think of for consumption. Each child completed brainstorming separately with supervision from a trained research assistant, who collected the responses on a laptop computer using the Concept System® Global MAX™ web-based platform. Brainstorming took approximately 3–5 minutes per participant. Saturation was reached after 51 participants completed the activity, at which point 121 reasons for caffeinated SD consumption had been reported and no new reasons were generated. The original list of 121 reasons was condensed using idea synthesis, a form of qualitative content analysis that combines redundant ideas to create a condensed list of independent reasons using the participants' original wording (36, 37). Idea synthesis resulted in a final list of 58 reasons, which were edited for syntactic consistency and represented the original set of reasons for caffeinated SD consumption reported by the participants.

### Sorting

For sorting, the 58 reasons were printed and laminated onto cards so that each child (*n* = 70) could manually sort each of the reasons (generated during brainstorming) into piles based on their perceived meaning. Prior to beginning the sorting activity, a trained research assistant (RA) presented each child with the stack of 58 cards, each containing a single reason, and instructed them to individually sort each reason into mutually exclusive piles in a way that made sense to them. Children were instructed not to (1) create piles such as “Miscellaneous” or “Other;” (2) sort reasons by personal relevance; or (3) leave any reasons unsorted. Children were also asked to name each of the piles to reflect their collective meaning, even if a pile contained only one card. The sorting activity typically lasted between 25 and 35 minutes per child.

### Rating

For rating, each child (*n* = 74) completed a paper survey, administered by a trained RA, on which they were instructed to rate on a five-point Likert-style scale (0 = not at all important to 5 = extremely important) the relative importance of each of the 58 reasons for their consumption of caffeinated SDs. Rating took approximately 10 minutes per participant.

### Statistical Analysis

The sorting and rating data were entered into the Concept System® Global MAX™ web-based platform, after which the data were analyzed in an iterative process (38, 39). First, multidimensional scaling (MDS) was used to a generate a point map, which was the basis for the subsequent concept maps, described below. The point map's goodness-of-fit was assessed using stress values. Stress values below 0.39 for MDS two-dimensional maps ensure a <1% probability of the matrix having a random structure or no structure (40). Based on a prior pooled analysis, the mean stress value for concept mapping studies is 0.28 (41). The SODA MAPS yielded a stress value of 0.25, indicative of a structured, non-random point map that represented the multivariate data collected, and thus was suitable for continued analyses and generation of subsequent concept maps (42).

Second, a hierarchical cluster analysis using Ward's algorithm was conducted to derive point-cluster maps. Cluster replay maps, which successively display cluster maps of fewer and fewer cluster solutions, were reviewed to determine which cluster maps offered the best conceptual fit of the data. Based on observation, cluster maps with a seven-, eight-, nine-, and ten-cluster solution appeared to be a better fit conceptually. These maps were then examined in greater detail, and points within each of the clusters on each map were carefully examined to ensure appropriate fit. Based on the conceptual meaning of each cluster, and the research team's expertise and prior qualitative findings related to children's SD intake (33, 43), it was determined that the eight-cluster map provided the best fit. Specifically, the eight-cluster map removed the need for themes to be unnecessarily divided (e.g., two energy clusters) and most clearly represented the participants' conceptualization of their caffeinated SD consumption.

Spanning analysis was then conducted, and bridging indices (BI) were calculated to examine the degree to which each point was an anchor on the eight-cluster map or a bridge to other thematic content (36, 38). The BI values reflect whether a reason was generally sorted with other nearby reasons (values closer to 0) or with items located further away on the concept map (values closer to 1). Based on these quantitative analyses (SEH, AJV, ACS) and expert judgement (AJV, ACS), cluster lines were redrawn to reflect optimal conceptual fit, resulting in the redistribution of 21 reasons to the closest adjacent cluster without altering each reason's original location on the map (38). Once the eight-cluster map was finalized, cluster names were generated using the original pile names provided by participants during the sorting activity.

Third, mean cluster rating values, computed from the mean rating values of each reason within a cluster, were added to create three-dimensional cluster rating maps.

## Results

Characteristics of participants in brainstorming (*n* = 51) and sorting/rating (*n* = 77) are shown in Table 1. The mean rating values for the 58 reasons for caffeinated SD consumption are shown in Table 2. The highest rated reasons included: “They taste good,” “They have good flavor,” “They are sweet,” “They are good,” “I love drinking them,” “They are my favorite drinks,” and “They are good for parties.”

**Table 1:** Participant characteristics for brainstorming and sorting/rating.

	**Brainstorming**	**Sorting/Rating**
N	51	77^a^^,^ ^b^
Age (mean ± SD)	10.7 ± 2.0	10.6 ± 1.8
**Sex (*****n*****, %)**		
Male	31, 61%	41, 53%
Female	20, 39%	36, 47%
**Race/Ethnicity (*****n*****, %)^c^**		
Non-Hispanic Black	31, 61%	56, 73%
Hispanic	10, 20%	12, 16%
Non-Hispanic White	6, 12%	
Other	4, 8%	9, 12%

a *n = 7 completed rating and not sorting*.

b *n = 3 completed sorting and not rating*.

c *Percentages do not add up to 100% due to rounding*.

**Table 2:** Rank ordering of reasons for caffeinated sugary drink intake based on rating values.^a^

**Ranking**	**Reason (reason number)**	**Mean Rating Values**
1	They taste good (19)	4.76 ± 0.59
2	They have good flavor (16)	4.54 ± 0.81
3	They are sweet (58)	4.51 ± 0.86
4	They are good (56)	4.41 ± 1.03
5	I love drinking them (11)	4.30 ± 1.11
6	They are my favorite drinks (26)	4.07 ± 1.31
7	They are good for parties (10)	4.00 ± 1.37
8	They have a nice aftertaste (13)	3.93 ± 1.20
9	I am thirsty (3)	3.88 ± 1.26
10	There are different types of flavors (33)	3.84 ± 1.43
11	I need something sweet (17)	3.78 ± 1.08
12	They are refreshing (1)	3.74 ± 1.18
13	They give me energy (41)	3.68 ± 1.52
14	They make me hype (44)	3.55 ± 1.57
15	They have lots of sugar (28)	3.51 ± 1.42
16	It is hot out (53)	3.49 ± 1.63
17	I like them on road trips (25)	3.38 ± 1.59
18	The sweetness is addictive (39)	3.34 ± 1.57
19	I need a boost (35)	3.32 ± 1.61
20	They are better than other drinks (37)	3.23 ± 1.53
21	Kids like them (54)	3.18 ± 1.72
22	My energy is low (38)	3.05 ± 1.63
23	I like them better than water (22)	3.00 ± 1.64
24	They are filling (9)	2.99 ± 1.40
25	They keep me awake (12)	2.99 ± 1.58
26	Water does not have a taste (57)	2.99 ± 1.70
27	My family drinks them (14)	2.97 ± 1.58
28	I cannot stop drinking them (29)	2.95 ± 1.57
29	They help me play (24)	2.92 ± 1.62
30	They are fizzy (27)	2.92 ± 1.62
31	They make me ready for a hard day (51)	2.86 ± 1.62
32	They are fruity (7)	2.78 ± 1.61
33	There is not water available (55)	2.77 ± 1.56
34	They help me concentrate (52)	2.72 ± 1.66
35	They give me a sugar rush (21)	2.72 ± 1.53
36	They are caffeinated (40)	2.69 ± 1.59
37	They are like coffee for kids (48)	2.68 ± 1.71
38	There may not be juice available (4)	2.66 ± 1.32
39	I need to calm down when I am angry (46)	2.65 ± 1.66
40	They wake me up (18)	2.64 ± 1.52
41	I like the bubbles (49)	2.51 ± 1.56
42	There is nothing else I like to drink (42)	2.47 ± 1.57
43	They make me run faster (36)	2.41 ± 1.50
44	They make me burp so my stomachache goes away (43)	2.38 ± 1.65
45	They get rid of burning from eating spicy food (15)	2.34 ± 1.58
46	I like the acid (47)	2.30 ± 1.56
47	I am bored (23)	2.27 ± 1.49
48	I want to burp (45)	2.24 ± 1.54
49	They make me ready to go to school (32)	2.19 ± 1.51
50	I do not like water (34)	2.19 ± 1.53
51	They keep me the perfect size (2)	2.18 ± 1.36
52	They are sour (5)	1.90 ± 1.26
53	I am sleepy (6)	1.89 ± 1.32
54	They make my headache go away (8)	1.85 ± 1.31
55	They keep me in shape (50)	1.82 ± 1.30
56	They are healthy for you (30)	1.61 ± 0.98
57	They help with my cramps (20)	1.55 ± 1.11
58	They make me smart (31)	1.55 ± 1.09

a *Values are means ± SDs. Each of the numbers in parentheses after each reason is the identifying number on the point map and point-cluster map; these numbers do not signify any value. Mean rating values ranged from a low of 1 (not at all important) to 5 (extremely important)*.

**Figure 1:**
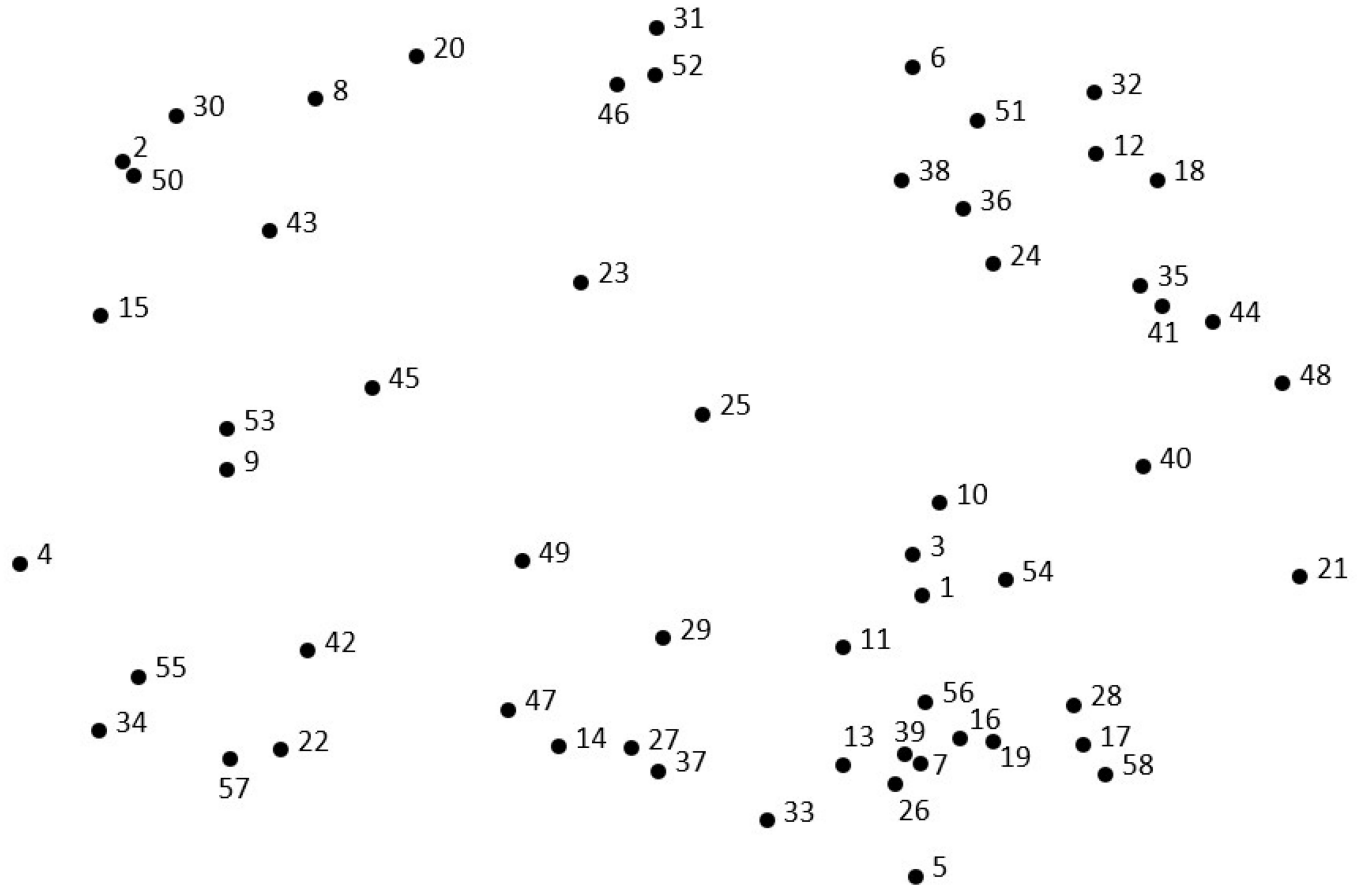
Point map of the 58 reasons for caffeinated SD consumption. Each point represents 1 of the 58 reasons that were brainstormed and sorted by the participants. Point location is an indicator of that point's relation to all other points; points located closer together were conceptually grouped together more frequently than points located distally. The numbers that appear next to each point on the map are not an indication of quantitative value, but instead serve to identify each specific reason (randomly assigned).

**Figure 2:**
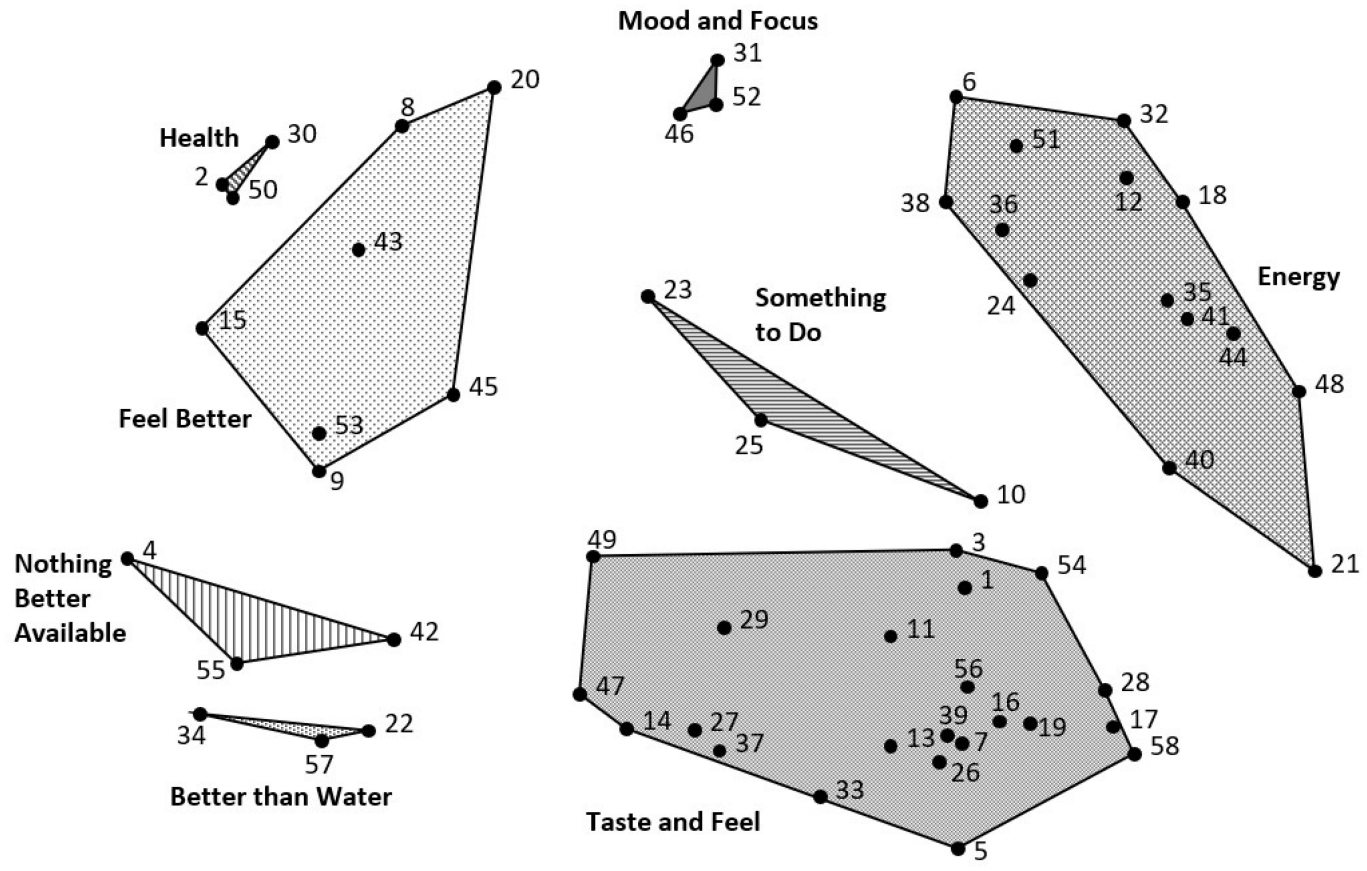
Point-cluster map of caffeinated SD consumption. Each of the eight clusters indicates a dimension of thematically similar content, conceptually grouped together from the 58 reasons for consumption. The clusters include *Health, Mood and Focus, Something to Do, Energy, Taste and Feel, Nothing Better Available, Better than Water*, and *Feel Better*. ^1^The cluster names reflect the names provided by the participants when sorting the reasons into piles.

The point map (Figure 1) represents the inter-relatedness of the 58 reasons for caffeinated SD consumption. The relative proximity of the reasons reflected their perceived similarity during the sorting activity. Reasons frequently sorted together were located closer together on the point map, while reasons sorted together infrequently were located further apart. Among the eight clusters (Figure 2), the clusters with the lowest BI values, indicating more narrowly focused thematic content, were *Taste and Feel* (0.19), *Something to Do* (0.36), and *Energy* (0.5), as illustrated by the relatively compressed shapes on the cluster map. The mean BI for each cluster is shown in Table 3.

**Table 3:** Rating and bridging indices for the 58 reasons for caffeinated sugary drink consumption by cluster.

**Cluster**	**No.^a^**	**Reasons**	**Rating value**	**Bridging value**
Taste and Feel			3.52	0.19
	19	They taste good	4.76	0.05
	16	They have good flavor	4.54	0.02
	58	They are sweet	4.51	0.21
	56	They are good	4.41	0.04
	11	I love drinking them	4.30	0.02
	26	They are my favorite drinks	4.07	0.06
	13	They have a nice aftertaste	3.93	0.00
	3	I am thirsty	3.88	0.20
	33	There are different types of flavors	3.84	0.22
	17	I need something sweet	3.78	0.28
	1	They are refreshing	3.74	0.09
	28	They have lots of sugar	3.51	0.22
	39	The sweetness is addictive	3.34	0.10
	37	They are better than other drinks	3.23	0.26
	54	Kids like them	3.18	0.27
	14	My family drinks them	2.97	0.37
	29	I cannot stop drinking them	2.95	0.22
	27	They are fizzy	2.92	0.27
	7	They are fruity	2.78	0.03
	49	I like the bubbles	2.51	0.33
	47	I like the acid	2.30	0.41
	5	They are sour	1.90	0.46
Something to Do			3.22	0.36
	10	They are good for parties	4.00	0.19
	25	I like them on road trips	3.38	0.34
	23	I am bored	2.27	0.53
Energy			2.83	0.50
	41	They give me energy	3.68	0.40
	44	They make me hype	3.55	0.47
	35	I need a boost	3.32	0.40
	38	My energy is low	3.05	0.46
	12	They keep me awake	2.99	0.43
	24	They help me play	2.92	0.38
	51	They make me ready for a hard day	2.86	0.47
	21	They give me a sugar rush	2.72	0.67
	40	They are caffeinated	2.69	0.47
	48	They are like coffee for kids	2.68	0.71
	18	They wake me up	2.64	0.46
	36	They make me run faster	2.41	0.43
	32	They make me ready to go to school	2.19	0.59
	6	I am sleepy	1.89	0.62
Better than Water			2.73	0.80
	22	I like them better than water	3.00	0.69
	57	Water does not have a taste	2.99	0.77
	34	I do not like water	2.19	0.94
Nothing Better Available			2.64	0.76
	55	There is not water available	2.77	0.75
	4	There may not be juice available	2.66	0.98
	42	There is nothing else I like to drink	2.47	0.54
Feel Better			2.41	0.73
	53	It is hot out	3.49	0.80
	9	They are filling	2.99	0.82
	43	They make me burp so my stomachache goes away	2.38	0.65
	15	They get rid of burning from eating spicy food	2.34	1.00
	45	I want to burp	2.24	0.53
	8	They make my headache go away	1.85	0.66
	20	They help with my cramps	1.55	0.66
Mood and Focus			2.31	0.66
	52	They help me concentrate	2.72	0.58
	46	I need to calm down when I am angry	2.65	0.69
	31	They make me smart	1.55	0.70
Health			1.87	0.77
	2	They keep me the perfect size	2.18	0.77
	50	They keep me in shape	1.82	0.78
	30	They are healthy for you	1.61	0.76

a *The numbers in the column left of the reasons serve to identify each reason as identified on the point map and are randomly assigned. These numbers do not indicate quantitative value*.

**Figure 3:**
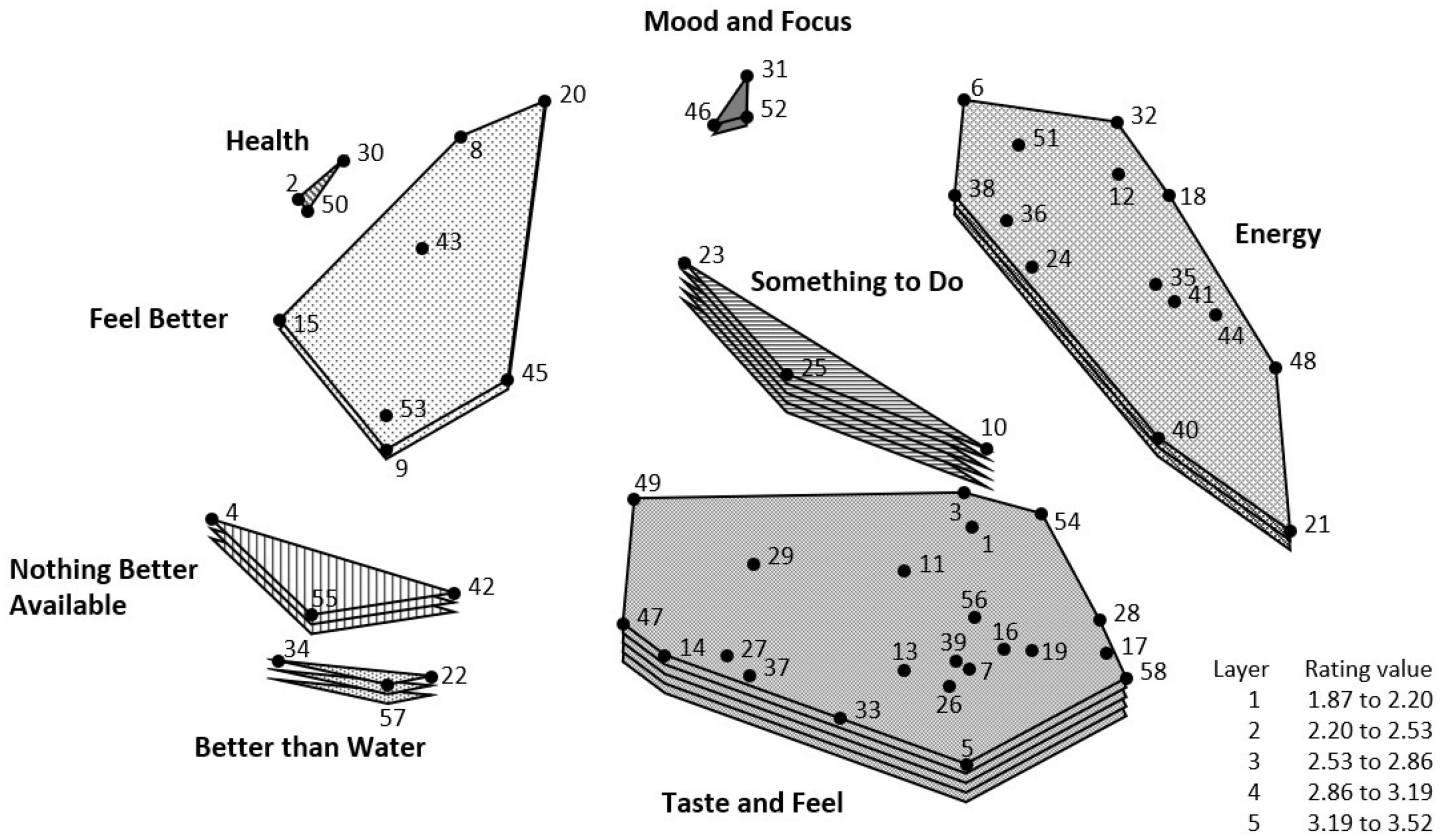
Cluster-rating map of reasons for caffeinated SD consumption. The cluster-rating map illustrates the mean importance rating influencing consumption for each cluster; clusters with a greater number of layers were rated as more important to participants' consumption. The top three rated factors (in order from highest to lowest) include *Taste and Feel, Something to Do*, and *Energy*.

The three-dimensional cluster rating map, based on the mean of the mean of the participants' ratings of each reason within a cluster, is shown in Figure 3. Mean cluster ratings are represented by a layering system; the greater the number of layers, the higher the mean cluster rating. The three highest rated clusters were *Taste and Feel* (3.52), *Something to Do* (3.22), and *Energy* (2.83).

## Discussion

In this study, children informed the development of a participant-driven conceptual framework (SODA MAPS) that provides a comprehensive understanding of the reasons for their caffeinated SD consumption. This framework, developed through participants brainstorming, sorting, and rating 58 distinct reasons for caffeinated SD intake, offers a unique and more nuanced conceptualization of children's caffeinated SD intake behaviors, as compared with prior studies (33, 44).

The findings demonstrate that children consume caffeinated SDs for a variety of reasons, the most influential being related to the drinks' palatability. This is demonstrated by the *Taste and Feel* cluster (which contained reasons such as “They taste good” and “They are sweet”) having the highest rating. This finding is unsurprising, as caffeinated SDs contain large quantities of added sugars (e.g., a 12-oz Coca-Cola^TM^ contains 39 g of sugar), and children report a heightened preference for sweetness compared with adults (45–48). In addition to high added sugar content, other reported reasons for caffeinated SD consumption within the *Taste and Feel* cluster pertained to common drink properties, including carbonation (e.g., “I like the bubbles”) and acidity (e.g., “I like the acid”).

Reasons reported within the cluster *Better than Water* (e.g., “Water does not have a taste”) also relate to palatability, and as such, were located in close proximity to the *Taste and Feel* cluster on the SODA MAPS. While most children reported liking water in a prior study with a demographically similar sample of children 8–14 years old (33), the higher perceived palatability of SDs relative to water emphasizes the need to take further actions to limit children's access to SDs. This finding also supports ongoing public health campaigns to offer children water in place of SDs whenever possible (49), consistent with the concept of changing environmental conditions to promote the selection of “optimal defaults” (50).

Another key finding was that, consistent with our recent qualitative findings (33), children described perceived increases in energy as a key reason for their caffeinated SD consumption. While there are previous reports of child hyperactivity resulting from caffeinated SD intake (33), the deliberate use of SDs to achieve a desired outcome, as demonstrated by reasons within the *Energy* cluster such as “They help me stay awake” and “They make me ready for a hard day,” suggests that children's caffeinated SD consumption behaviors may parallel well-described behavioral patterns surrounding coffee consumption in adults (51). The purposeful consumption of SDs also reflects established patterns of caffeine use in adolescents (52). Use of caffeinated SDs to boost energy may also suggest that children and adolescents get inadequate sleep, perhaps as a result of excess screen time (53). While our study design did not allow us to distinguish whether reported reasons for caffeinated SD intake were due to their sugar content, caffeine content, or both, our findings highlight the need to investigate the likelihood that sugar and caffeine in SDs may independently and synergistically promote their continued consumption. This is consistent with recent evidence demonstrating that some children may become physically and/or psychologically dependent on caffeinated SDs (54, 55).

While reasons within the *Mood and Focus* and *Feel Better* clusters were not rated as highly compared to those within the *Taste and Feel* or *Energy* clusters, children also reported reasons for caffeinated SD intake related to affective regulation (e.g., “I need to calm down when I am angry”). Withdrawal-like symptoms, both affective (e.g., irritability, sadness) and physical (e.g., headache, stomachache), have been previously reported among children in response to highly processed foods (56–58). Additionally, abstinence from habitual caffeine doses as low as 100 mg per day (comparable to the amount found in two cans of caffeinated soda) has been shown to induce withdrawal symptoms (e.g., headaches) in adults (59). Thus, reasons for children's caffeinated SD intake within *Mood and Focus* and *Feel Better* may reflect important and currently overlooked barriers to sustained reduction in children's caffeinated SD intakes.

While the majority of the reasons for SD consumption reported in the present study were at the individual level, children's dietary behaviors are also strongly influenced by environmental and situational factors (60), such as the availability and accessibility of SDs relative to alternative beverages (61). The cluster *Nothing Better Available* calls attention to environmental and community influences (62, 63), which may be particularly critical in urban, low-income communities, where access to fast food and junk food is often high relative to healthier options (64–67). Furthermore, reasons within the *Something to Do* cluster call attention to the importance of normative behaviors (e.g., “Good for parties,” “I like them on road trips”) in influencing children's caffeinated SD intake. Consumption of SDs as a means of alleviating boredom, for example, also suggests that encouraging participation in activities, such as afterschool programs or youth sports, may help to reduce children's caffeinated SD intake. Furthermore, provision of unsweetened, carbonated beverages, such as flavored seltzer water, instead of plain water, may offer a healthy and “less boring” substitute for caffeinated SDs. The influence of cultural and social norms is well described for other dietary behaviors among children (33, 68, 69), and altering norms surrounding risk behaviors has shown promise in initiating lifestyle behavior change among children (70–72).

As the first study to use concept mapping to elucidate reasons for children's caffeinated SD intake, SODA MAPS provide a novel framework for conceptualizing the multifactorial reasons for children's caffeinated SD consumption. The use of concept mapping methodology allowed for the quantitative and qualitative evaluation of the reasons for children's caffeinated SD consumption. However, while the results of this study provide novel insights into caffeinated SD consumption among children, the analysis was subject to several limitations. The sample population was geographically limited (all recruited from Washington, D.C.), as well as racially/ethnically limited (primarily non-Hispanic Black and Hispanic participants). While these could be viewed as strengths, especially given the well-documented disparities in SD consumption and related cardiometabolic health outcomes in minority populations (13), our sample is not representative of all children who consume caffeinated SDs. In addition, selection bias may have affected the makeup of the study population, as it was a convenience sample. Intakes of other, non-beverage, sources of caffeine (e.g., chocolate, dietary supplements), which may influence reasons for children's caffeinated SD consumption, were also not evaluated.

The findings of this study provide a comprehensive conceptual framework for understanding children's caffeinated SD consumption, which is encouraged by a variety of biological (e.g., *Energy*), psychological (e.g., *Mood and Focus*), normative (e.g., *Something to Do*), and environmental factors (e.g., *Nothing Better Available*), as well as the palatability of caffeinated SDs (e.g., *Taste and Feel*). Collectively, these findings support the need for multi-level interventions aimed at addressing individual, sociocultural, and environmental influences on children's SD intake and contribute to informing the development of tailored interventions to reduce SD consumption among children.

## Data Availability Statement

The raw data supporting the conclusions of this article will be made available by the authors, without undue reservation.

## Ethics Statement

The studies involving human participants were reviewed and approved by the Institutional Review Board at The George Washington University [Protocol 18091], and the Institutional Review Board at Children's National Hospital [Protocol 00011014]. Written informed consent to participate in this study was provided by the participants' legal guardian/next of kin.

## Author Contributions

ACS, AJV, and JS designed the research. SEH performed the analyses. SEH, ACS, and AJV interpreted the data. SEH wrote the first draft of the manuscript. All authors were involved in editing the manuscript and approved the final version.

## Conflict of Interest

The authors declare that the research was conducted in the absence of any commercial or financial relationships that could be construed as a potential conflict of interest.
